# Supplementary material for: Composition, determinants, and risk factors of low birth weight in Sri Lanka
Source: PLoS One. 2025 Feb 7;20(2):e0318554. doi: 10.1371/journal.pone.0318554 (PMC11805389; doi:10.1371/journal.pone.0318554)
Supplement: S1 File — (PDF) [file pone.0318554.s001.pdf]

Supplemental Figures and Tables

Manuscript title: Composition, determinants, and risk factors of low birth weight in Sri Lanka

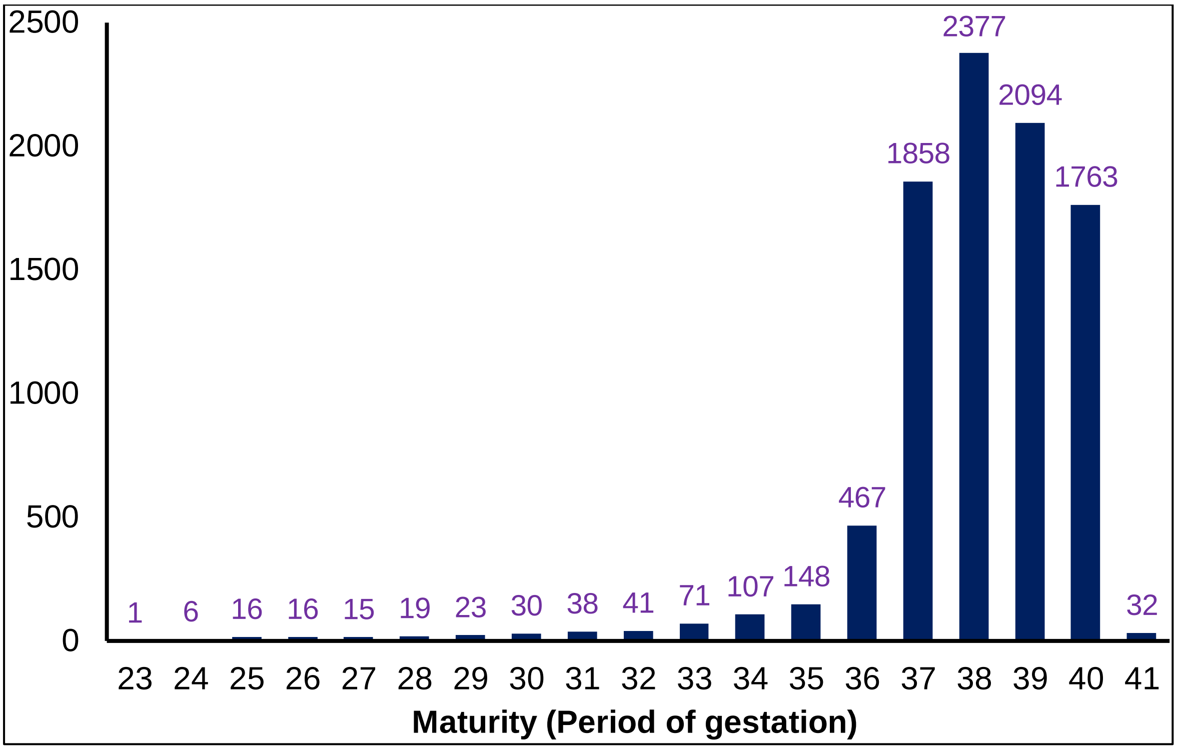

Supplemental Figure 1 – Number of neonates born at each gestation

Supplemental Table 1 – Number of neonates recruited from each study site

**Study site**	**Number of live births**	**Percentage**
BH Balangoda	240	2.6%
BH Gampola	350	3.8%
BH Puttalam	735	8.1%
CNTH Ragama (TH)	744	8.1%
DGH Kegalle	351	3.8%
DGH Nuwara Eliya	508	5.6%
DMH Colombo (TH)	1051	11.5
BH Kethumathi	386	4.2%
PGH Badulla	738	8.1%
TH Anuradhapura	966	10.6%
TH Batticaloa	877	9.6%
TH Jaffna	1058	11.6%
TH Mahamodara	1126	12.3%
**Total**	**9130**	**100.0%**

Supplemental Table 2 – Gender distribution of the study population

**Study site**	**Male**		**Female**	
	N	***%***	***N***	***%***
BH Balangoda	119	49.6%	121	50.4%
BH Gampola	156	44.6%	194	55.4%
BH Puttalam	384	52.2%	351	47.8%
CNTH Ragama (TH)	391	52.6%	353	47.4%
DGH Kegalle	168	48.0%	182	52.0%
DGH Nuwara Eliya	254	50.0%	254	50.0%
DMH Colombo (TH)	565	53.8%	485	46.2%
BH Kethumathi	185	47.9%	201	52.1%
PGH Badulla	383	51.9%	355	48.1%
TH Anuradhapura	505	52.3%	461	47.7%
TH Batticaloa	474	54.1%	402	45.9%
TH Jaffna	568	53.7%	489	46.3%
TH Mahamodara	626	55.7%	497	44.3%
**Total**	**4778**	**52.4%**	**4345**	**47.6%**

** 7 babies whose gender could not be determined at birth were excluded from the table.*

Supplemental Table 3 – Birth weight distribution in each study site

	N	Mean birth weight (g)	SD of birth weight (g)	Median birth weight (g)	Birth weight range (g)
BH Balangoda	237	2830	467	2810	1340-4340
BH Gampola	350	2803	455	2800	635-4150
BH Puttalam	734	2901	478	2890	640-4520
CNTH Ragama (TH)	744	2795	593	2875	410-4165
DGH Kegalle	351	2751	473	2780	550-4580
DGH Nuwara Eliya	508	2706	447	2750	690-4020
DMH Colombo (TH)	1051	2821	546	2860	489-4820
BH Kethumathi	386	2858	482	2877	755-4590
PGH Badulla	738	2763	524	2810	680-4330
TH Anuradhapura	966	2836	516	2890	470-4420
TH Batticaloa	877	2828	512	2865	470-4500
TH Jaffna	1057	2911	502	2950	730-4700
TH Mahamodara	1126	2838	493	2880	550-4280
**Total**	**9125**	**2827**	**512**	**2860**	**410-4820**

** 5 babies whose birth weight was not measured were excluded from the table.*

Supplemental Table 4 – SGA prevalence at each gestation

Gestation (weeks)	Total number of births of which SGA could be determined	Number of SGA births	Prevalence of SGA
24	6	0	0.0%
25	15	0	0.0%
26	16	6	37.5%
27	15	3	20.0%
28	19	3	15.8%
29	23	7	30.4%
30	30	8	26.7%
31	38	8	21.1%
32	41	7	17.1%
33	71	11	15.5%
34	107	20	18.7%
35	148	37	25.0%
36	467	104	22.3%
37	1855	369	19.9%
38	2376	415	17.5%
39	2093	383	18.3%
40	1763	424	24.0%
41	32	14	43.8%
**Total**	**9115**	**1819**	**20.0%**

Supplemental Table 5 – Mode of delivery of LBW, Preterm and SGA neonates

Mode of delivery	LBW (n=1865)	Preterm (n=998)	SGA (n=1819)	Total (n=9130)
Normal vaginal delivery	820 (44.0%)	367 (36.8%)	976 (53.7%)	5064 (55.5%)
Forceps delivery	10 (0.5%)	6 (0.6%)	21 (1.2%)	90 (1.0%)
Vacuum delivery	7 (0.4%)	0 (0%)	15 (0.8%)	90 (1.0%)
Elective caesarean section	447 (24.0%)	193 (19.3%)	354 (19.5%)	1929 (21.1%)
Emergency caesarean section	581 (31.2%)	432 (43.3%)	453 (24.9%)	1957 (21.4%)
