# Supplementary material for: Composition, determinants, and risk factors of low birth weight in Sri Lanka
Source: PLoS One. 2025 Feb 7;20(2):e0318554. doi: 10.1371/journal.pone.0318554 (PMC11805389; doi:10.1371/journal.pone.0318554)
Supplement: Attachment — Submitted filename: Responses for reviewer comments.docx [file pone.0318554.s003.docx]

**Responses to reviewer comments**

**Manuscript title:**	Composition, determinants, and risk factors of low birth weight in Sri Lanka

**Submission ID:**	PONE-D-24-07451

**Responses to Reviewer 1 Comments:**

1. This study provides important findings in the field of obstetrics and neonatal health. Accept for publication.

**Author response:** Thank you very much for this positive comment.

2. Revise and proofread the abstract, along with incorporating the results of P-values to strengthen the presentation.

**Author response:** We have revised the abstract by proofreading and incorporating the results of P-values as suggested.

**Responses to Reviewer 2 Comments:**

1. The cross sectional study is a valuable guide to the factors that contribute to low birth weight in Sri Lanka.

**Author response:** Thank you very much for this positive comment.

2. The authors refer to “small for gestational age” as a “cause” of low birth weight in the Introduction, but it is really a category, not a cause. The authors could begin by briefly describing the likely predominant causes of babies that are born SGA, which they analyze in detail during the Results and Discussion.

**Author response:** Thank you very much for the suggestion. We have revised the introduction as suggested.

3. Authors’ characterize the dataset from Sri Lanka as being unique among South Asian countries because of the high rate of births in health care facilities, but they cite no direct measurement from other South Asian countries.

**Author response:** We have revised the introduction by including institutional delivery rates of other South Asian countries.

4. There is no consideration of the high “elective” Cesarean-section rate among study subjects on birth weight, which has been shown in some studies to increase LBW.

**Author response:** We have included a new Supplementary Table (Supplementary Table 5) showing mode of delivery of LBW, Preterm and SGA neonates.

5. Authors found that Tamil ethnicity was an independent predictor for LBW. Were they able to determine whether nutritional status of the mothers was the major driver? Given the disparity in socioeconomic status between Tamil and Sinhalese Sri Lankans, this seems to be an obvious consideration.

**Author response:** We included the variables like maternal pre-pregnancy BMI and weight gain in pregnancy that evaluate the nutrition status in the regression analysis. Indian Tamil ethnicity, maternal pre-pregnancy BMI, and maternal weight gain in pregnancy were all independent predictors of SGA indicating that the Indian Tamil ethnicity is a predictor independent of nutritional status.
