# Supplementary material for: Composition, determinants, and risk factors of low birth weight in Sri Lanka
Source: PLoS One. 2025 Feb 7;20(2):e0318554. doi: 10.1371/journal.pone.0318554 (PMC11805389; doi:10.1371/journal.pone.0318554)
Supplement: Attachment — Submitted filename: Responses for reviewer comments.pdf [file pone.0318554.s004.pdf]

Responses to reviewer comments

Manuscript title: Composition, determinants, and risk factors of low birth weight in Sri Lanka

Submission ID: PONE-D-24-07451R1

Responses to Reviewer 1 Comments:

There were no comments, as this reviewer had already suggested accepting the manuscript at the first revision.

Responses to Reviewer 2 Comments:

1. Since the elective c-section rate reported is 20% nearly double the rate reported in high-resource countries, and delivery by c-section is associated with LBW, the authors should analyse whether or not elective c-section was an independent risk factor, or at a minimum acknowledge that this is a limitation.

   Author response: We agree that the elective caesarean section rate is higher than in the high-resource countries, but it is comparable with the country-specific data. We also agree that elective caesarean section could be associated with LBW. In the study, we did not examine the risk factors for LBW as a whole but examined the risk factors for SGA and prematurity separately. As elective caesarean sections are performed for already determined fetal growth restriction in utero and other indications requiring preterm delivery, elective caesarean section was not included in the regression models evaluating for independent risk factors of SGA and prematurity. We have acknowledged the higher rate of elective caesarean section as a limitation of the study, as suggested by the reviewer (Line 325-330).

Responses to Reviewer 3 Comments:

2. The bottom line for me is that this study has not added any information that we do not previously know.

   Author response: Thank you for your comment. This study is one of the most extensive country-wide studies evaluating the composition and determinants of low birth weight. It describes the relative contribution of prematurity and SGA for LBW in a developing country. It highlights that SGA contributes to the major proportion of LBW in developing countries. Although risk factors for prematurity and LBW are well established, the independent risk factors for SGA determined through large-scale country-wide studies in developing countries are lacking. This study fills that gap by describing the independent risk factors for SGA. We have improved the discussion highlighting this (Line 276-279).

Responses to Reviewer 4 Comments:

3. Neonatal morbidity and mortality remain high in developing countries. This study analysed risk factors for preterm birth in developing countries and measures that can be improved, and the sample size was large. The overall study design is reasonable, and the conclusions are reliable. It has important clinical value for reducing neonatal morbidity and mortality in developing countries and also provides measures for reducing neonatal morbidity and mortality. The authors responded in detail to the comments of the reviewers.

   Author response: Thank you very much for the positive comment.
